# Impact of Self-Reported Loss of Balance and Gait Disturbance on Outcomes following Adult Spinal Deformity Surgery

**DOI:** 10.3390/jcm13082202

**Published:** 2024-04-11

**Authors:** Bassel G. Diebo, Daniel Alsoof, Renaud Lafage, Mohammad Daher, Mariah Balmaceno-Criss, Peter G. Passias, Christopher P. Ames, Christopher I. Shaffrey, Douglas C. Burton, Vedat Deviren, Breton G. Line, Alex Soroceanu, David Kojo Hamilton, Eric O. Klineberg, Gregory M. Mundis, Han Jo Kim, Jeffrey L. Gum, Justin S. Smith, Juan S. Uribe, Khaled M. Kebaish, Munish C. Gupta, Pierce D. Nunley, Robert K. Eastlack, Richard Hostin, Themistocles S. Protopsaltis, Lawrence G. Lenke, Robert A. Hart, Frank J. Schwab, Shay Bess, Virginie Lafage, Alan H. Daniels

**Affiliations:** 1 Department of Orthopedics, Warren Alpert Medical School of Brown University, East Providence, RI 02914, USA; dr.basseldiebo@gmail.com (B.G.D.); daniel_alsoof@brown.edu (D.A.); mohdaher1@hotmail.com (M.D.); mariah_balmaceno-criss@brown.edu (M.B.-C.); 2 Department of Orthopedic Surgery, Lenox Hill Northwell, New York, NY 10075, USA; renaud.lafage@gmail.com (R.L.); fschwab@att.net (F.J.S.); virginie.lafage@gmail.com (V.L.); 3 Department of Orthopedics, NYU Langone Orthopedic Hospital, New York, NY 10016, USA; pgpassias@yahoo.com (P.G.P.); tprotopsaltis@gmail.com (T.S.P.); 4 Department of Neurosurgery, University of California, San Francisco, CA 94115, USA; christopher.ames@ucsf.edu (C.P.A.); vedat.deviren@ucsf.edu (V.D.); 5 Department of Neurosurgery, Duke Spine Division, Durham, NC 27708, USA; christopher.shaffrey@duke.edu; 6 Department of Orthopaedic Surgery, University of Kansas Medical Center, 3901 Rainbow Blvd., Kansas City, KS 66160, USA; dburton@kumc.edu; 7 Denver International Spine Center, Denver, CO 80218, USA; breton.line@gmail.com (B.G.L.); shay_bess@hotmail.com (S.B.); 8 Department of Orthopedic Surgery, University of Calgary, Calgary, AB T2N 1N4, Canada; alex.soroceanu@hotmail.com; 9 Department of Neurological Surgery, University of Pittsburgh, Pittsburgh, PA 15260, USA; kojohamilton1@gmail.com; 10 Department of Orthopaedic Surgery, University of California, 1 Shields Ave., Davis, CA 95616, USA; eric.klineberg@gmail.com; 11 San Diego Spine, La Jolla, CA 92037, USA; gmundis1@gmail.com (G.M.M.); reastlack@gmail.com (R.K.E.); 12 Hospital for Special Surgery, New York, NY 10021, USA; hanjokimmd@gmail.com; 13 Leatherman Spine Center, Louisville, KY 40202, USA; jlgum001@gmail.com; 14 Department of Neurosurgery, University of Virginia, Charlottesville, VA 22903, USA; jss7f@uvahealth.org; 15 Department of Neurosurgery, Barrow Neurological Institute, St. Joseph’s Hospital and Medical Center, Phoenix, AZ 85013, USA; juansuribe@gmail.com; 16 Johns Hopkins University School of Medicine, Baltimore, MD 21218, USA; kkebais@jhmi.edu; 17 Department of Orthopedics, Washington University in St Louis, St. Louis, MO 63110, USA; munishgupta@wustl.edu; 18 Spine Institute of Louisiana, Shreveport, LA 71101, USA; pnunley@louisianaspine.org; 19 Department of Orthopaedic Surgery, Baylor Scoliosis Center, 4708 Alliance Blvd #800, Plano, TX 75093, USA; rhostin@swscoli.com; 20 Department of Orthopedic Surgery, Columbia University Medical Center, The Spine Hospital at New York Presbyterian, New York, NY 10032, USA; lenkespine@gmail.com; 21 Swedish Neuroscience Institute, Seattle, WA 98027, USA; robert.hart@swedish.org

**Keywords:** balance, radiological outcomes, adult spinal deformity

## Abstract

**Background:** The objective of this study was to evaluate if imbalance influences complication rates, radiological outcomes, and patient-reported outcomes (PROMs) following adult spinal deformity (ASD) surgery. **Methods:** ASD patients with baseline and 2-year radiographic and PROMs were included. Patients were grouped according to whether they answered yes or no to a recent history of pre-operative loss of balance. The groups were propensity-matched by age, pelvic incidence–lumbar lordosis (PI-LL), and surgical invasiveness score. **Results:** In total, 212 patients were examined (106 in each group). Patients with gait imbalance had worse baseline PROM measures, including Oswestry disability index (45.2 vs. 36.6), SF-36 mental component score (44 vs. 51.8), and SF-36 physical component score (*p* < 0.001 for all). After 2 years, patients with gait imbalance had less pelvic tilt correction (−1.2 vs. −3.6°, *p* = 0.039) for a comparable PI-LL correction (−11.9 vs. −15.1°, *p* = 0.144). Gait imbalance patients had higher rates of radiographic proximal junctional kyphosis (PJK) (26.4% vs. 14.2%) and implant-related complications (47.2% vs. 34.0%). After controlling for age, baseline sagittal parameters, PI-LL correction, and comorbidities, patients with imbalance had 2.2-times-increased odds of PJK after 2 years. **Conclusions:** Patients with a self-reported loss of balance/unsteady gait have significantly worse PROMs and higher risk of PJK.

## 1. Introduction

The number of individuals aged 65 years and above is projected to reach 89 million in the United States by 2050 [1]. With an aging population, the prevalence of adult spinal deformity (ASD) is also predicted to rise. ASD is a disabling condition which results in low back pain, neurological dysfunction, and has also been associated with mental health disorders [2,3,4]. The association between ASD and disability has been demonstrated [5,6,7,8,9,10,11,12,13,14,15,16,17]. However, ASD patients can present with variable disabilities compared to what often corresponds to the type and severity of the spine deformity. To investigate the associations between disability and different spine deformity types, the International Spine Study Group (ISSG) performed a prospective, multi-center analysis on a population of approximately 500 ASD patients that had no history of spine surgery. Patients were organized into cohorts based upon spine deformity type and severity according to the Scoliosis Research Society-Schwab ASD classification [18]. The health-related impact of the specific spine deformity (measured by standard form version 2 score (SF-36), physical component score (PCS), and mental component score (MCS)) was compared to United States (U.S.) population norms and to other chronic diseases and disabilities, such as cardiac disease, diabetes, osteoarthritis, loss of vision, etc. [9]. The ISSG found that for all ASD patients, the impact that ASD had upon physical health was similar to that of the health impact of cardiac disease, cancer, and diabetes [9]. When ASD patients were divided into age cohorts, ASD patients in each cohort reported physical function levels that were below the 25th percentile for the U.S. population of the same age [9]. Additionally, as ASD patients aged, the decline in physical function for ASD patients was more rapid than that of the U.S. population of the same age (*p* < 0.05) [9]. These findings highlighted the massive impact that ASD can have upon physical health [9]. Importantly, however, when ASD patients were divided into specific deformity cohorts and compared to other chronic diseases, the impact upon physical function was found to be specific to each spine deformity type [9]. ASD patients with primarily thoracic scoliosis deformities had PCS scores similar to those reported by patients with chronic back pain [9]. Patients with primarily lumbar scoliosis deformities had PCS scores similar to those reported by patients with osteoarthritis and chronic heart disease [9]. Patients with primarily sagittal deformities (scoliosis < 20°, sagittal vertical axis (SVA) > 5 cm) reported PCS scores similar to patients with osteoarthritis and rheumatoid arthritis functioning below the 25th percentile [9]. Patients with severe sagittal deformity (SVA > 10 cm) had similar PCS scores as patients with chronic lung disease functioning below the 25th percentile [9]. Patients with combined lumbar scoliosis and sagittal malalignment reported severe disability with PCS scores worse than those reported by patients with limited vision (legally blind) and limited function of the arms and legs (amputees) [9]. These findings further highlighted the massive health impact that ASD can have upon patients, and provided comparative disease state and health impact analogies that are more readily understood by patients, the industry, and health care providers; namely, that ASD patients with severe deformities reported similar difficulties with physical function as patients who are amputees and as patients who are legally blind [9].

Surgical treatment for ASD has demonstrated reduced pain and improved health-related quality of life (HRQOL) compared to nonoperative treatment. In fact, Smith et al. preformed an analysis of ASD patients treated operatively vs. nonoperatively [19]. At minimum 2-year follow-up, all HRQOL measures improved for operative patients; however, nonoperative patients demonstrated only modest improvements in the Scoliosis Research Society-22r pain and satisfaction domains. Matched operative–nonoperative cohort analysis demonstrated that, at last follow-up, operative patients had better outcomes for all measures than nonoperative patients except short form-36 mental component scores. These data demonstrated that operative treatment for ASD can provide substantial improvement in HRQOL, whereas nonoperative treatment typically has little impact on patient-reported disability. However, surgical outcomes can be inconsistent. Recent data have demonstrated that many ASD patients remain poorly corrected following reconstructive surgery and are left with residual spinal malalignment, despite undergoing high-cost and high-risk surgical procedures [20,21].

A very important thing to assess in ASD patients is alignment, as this could affect the outcomes and increase the incidence of post-operative complications [22]. Furthermore, with aging, humans’ ability to coordinate and multitask deteriorates progressively as the degenerative process does not only involve the musculoskeletal but also the neurosensorial, motor, and cognitive systems. Therefore, functional assessment is becoming more and more important in order to assess the true impact of ASD on a patient’s functional capacity [23]. When standing, the body maintains its center of gravity over the feet while maintaining a horizontal gaze [24]. Patients with spinal deformity have deviations in the center of mass secondary to anterior truncal inclination in the sagittal plane, in addition to coronal and axial deformities. Therefore, the positions of the head and trunk in space are altered. Intuitively, spinal deformities that are driven by chronic degenerative disc and soft tissue cascades might alter the neurosensorial and proprioceptive input from the body mass to the brain over time [13]. In fact, Moustafa et al. reported that thoracic kyphosis is correlated with the deficiency of sensorimotor control [25,26]. Often neglected is that spinal realignment through recent strides in implants and technology provides rapid correction over a short operative duration. It remains unknown whether patients’ ability to adapt and coordinate their new realignment-driven neurosensorial input post-operatively is related to the variability in surgical outcomes.

As a proxy to patients’ coordination and balance, this study investigated patients who reported a history of fall or unsteady gait prior to their spinal realignment for ASD. The aim of this study was to evaluate if imbalance influences complication rates, radiological outcomes, and patient-reported outcome measures (PROMs) following ASD surgery in a matched cohort of patients. The hypothesis was that patients with reported imbalance had higher rates of complications and worse PROMs and radiographic outcomes.

## 2. Materials and Methods

### 2.1. Study Design

This was a retrospective cohort study of a multi-center prospective database across 13 spine centers in the United States. An Institutional Review Board approved this investigation (Institutional Name: HCA—HealthONE Institutional Review Board, Denver, Colorado. Approval Number: 231842-20). Participants provided informed consent and consent was written.

The study sample included adult spinal deformity patients who underwent a primary posterior long spinal fusion. Patients with a history of prior spine surgery were excluded. The fusion construct included an upper instrumented vertebra (UIV) at L1 and above, with the lower instrumented vertebra (LIV) at S1 or the ilium. Patients with baseline and 2-year post-operative radiological and patient-reported outcome measures (PROMs) were retained for analysis.

### 2.2. Variables

Patient demographic data were collected at baseline, including baseline frailty assessment (using the Edmonton frailty scale [27]). Patients were pre-operatively grouped into those who answered “Yes” or “No” to a recent history of loss of balance or gait instability. The groups were subsequently matched by age, baseline pelvic incidence–lumbar lordosis (PI-LL), and surgical invasiveness.

Outcomes of interest included the sagittal and coronal alignment parameters and PROMs. Radiological parameters included pelvic tilt (PT), PI-LL, sagittal vertical axis (SVA) T4-T12 kyphosis, T1 pelvic angle (T1PA), Upper Thoracic Cobb Angle, thoracic cobb angle, Thoracolumbar Cobb Angle, Lumbar Cobb Angle, and lumbosacral cobb angle [28,29]. The PROMs collected were the Oswestry disability index (ODI) [30], the SF-36 mental component score (SF-36 MCS), the SF-36 physical component score (SF-36 PCS) [31], and the SRS-22 total and activity, pain, appearance, mental, and satisfaction domain scores [32].

Post-operative complications and outcomes were collected, including re-operation, major complication, radiographical fusion after 2 years, proximal junctional kyphosis (PJK) after 2 years, and implant-related complications. Implant-related complications included interbody fusion dislocation, loose implants, painful implants, rod breakage, rod dislocation, screw breakage, screw loosening, and PJK.

### 2.3. Statistical Analysis

Data were recorded in a Microsoft Excel spreadsheet, then analyzed using SPSS (IBM SPSS Statistics, Version 27.0. Armonk, NY, USA: IBM Corp). Demographic data, complication rates, radiographical parameters, and PROMs were compared between the groups using independent *t*-tests and chi-squared tests. A binary logistic regression model controlling for age, baseline sagittal parameters, PI-LL correction, and the Charlson Comorbidity Index was utilized to investigate the impact of reporting a history of imbalance on sustaining post-operative PJK.

## 3. Results

### 3.1. Demographics

Before matching, 133 out of 267 patients reported loss of balance (49.81%). After matching, 212 patients were reported, with 106 patients in each group for analysis. The mean age (64 vs. 63 years), BMI (27.2 vs. 27.0 kg/m^2^), and gender (76% vs. 87% female) as well as median UIV/LIV were not significantly different for patients with imbalance and without imbalance, respectively (all *p* > 0.05). Patients in the imbalance group had a higher Frailty Index Score compared to patients without imbalance (3.74 vs. 2.33, *p* < 0.001) (Cohen’s d = 1.1) (Table 1).

### 3.2. Baseline Characteristics

At baseline, pelvic tilt (PT), pelvic incidence–lumbar lordosis (PI-LL), and Sagittal Vertical Alignment (SVA) were comparable. Patients with reported imbalance had a significantly lower thoracic cobb angle (25.3° vs. 37.5°, *p* < 0.001) and Lumbar Cobb Angle (37.4° vs. 45.5°, *p* = 0.004), although their global coronal alignment (C7 PLA) was similar (imbalance: 41.5 vs. 34.3 mm, *p* = 0.16) (Table 2).

At baseline, patients with imbalance had worse PROM measures, including ODI (45.2 vs. 36.6), SF-36 MCS (44 vs. 51.8), SF-36 PCS (30.2 vs. 35.1), and SRS-22 mental (3.3 vs. 3.8) (*p* < 0.001 for all) (Table 3).

After 2 years, patients with imbalance had less PT correction (−1.2° vs. −3.6°, *p* = 0.04) for a comparable PI-LL correction (−11.9° vs. −15.1°, *p* = 0.14). Patients with imbalance also had lower T1PA correction (−3.22° vs. −5.77°, *p* = 0.47). Both groups demonstrated similar improvements in their coronal plane deformity (Table 4).

Imbalance patients had higher rates of radiographic PJK (26.4% vs. 14.2%, *p* = 0.03) and implant-related complications (47.2% vs. 34.0%, *p* = 0.05). Imbalance patients also had a higher rate of proximal junctional failure (PJF), defined as a PJK angle difference greater than 20° or re-operation due to PJK (34.00% vs. 17.92%, *p* = 0.01). After controlling for age, baseline sagittal parameters, PI-LL correction, and CCI, patients with imbalance had 2.2-times-increased odds of sustaining PJK after 2 years (Table 5).

## 4. Discussion

The spine and sensorimotor system provide postural balance, which is critical in the prevention of falls and maintaining quality of life [8,33]. Godzik et al. found that, compared with healthy controls, ASD patients have diminished sensory integration with impaired postural stability and delayed response to external postural changes [34]. With a growing prevalence of ASD, clinicians need to have an easily accessible way of identifying how loss of balance impacts surgical outcomes to enable subsequent risk stratification. Our study demonstrated that self-reported loss of balance and unsteady gait is a proxy for frailty with worse PROMs. These patients were also demonstrated to have 2.2-fold-increased odds of PJK after 2 years. These results support our proposed hypothesis.

There are several shared characteristics between falls and frailty, with Ensrud et al. demonstrating that frailty is an independent predictor of falls [35]. In prior research on ASD patients, Godzik et al. evaluated postural stability and compared it with an age-matched cohort [34]. Patients with self-reported falls had a lower SRS-22 self-image score as compared to non-fallers. In fact, a systematic review by Laverdiere et al. showed that frailty correlated with the risk of blood transfusion, deep vein thrombosis, pulmonary embolism, mortality, PJK, PJF, and a higher SVA [36]. On the other hand, a low frailty index was associated with a lower rate of complications both pre-operatively and after 2 years [36]. The present study provides further insights into the association between loss of balance in ASD, quality of life metrics, and post-operative complications. Imbalanced patients had worse PROMs at baseline, including ODI, SF-36 PCS, and SF-36 MCS. Additionally, the imbalanced group of patients had a significantly higher Frailty Index Score than non-imbalanced patients. Therefore, asking patients if they have gait instability or loss of balance is a helpful representation of baseline quality of life and frailty, and may serve as a proxy for frailty.

Previous studies have established altered gait patterns in patients with ASD, which can include gait asymmetry, reduced stride length, and reduced stride velocity [37,38]. These factors can contribute to instability, loss of balance, and falls. However, surgical intervention with fusion constructs has been shown to improve gait mechanics. In patients undergoing primary or revision deformity surgery, Engsberg et al. concluded that surgery objectively improved gait endurance and speed [39,40]. One goal of reconstructive spinal surgery is to restore sagittal alignment, which may subsequently improve postural balance [13]. The current study reveals that patients who report imbalance have improved post-operative sagittal and coronal alignments. When comparing the balance and imbalance groups, patients who reported imbalance had less thoracic cobb angle correction, from baseline to 2-year follow-up, than patients who did not report imbalance. However, this likely represents the higher thoracic cobb angle of the balanced group at baseline. Imbalance patients also had less PT correction for a comparable PI-LL correction. This may reflect the failure to relax compensatory mechanisms in patients with imbalance. Although surgery is likely to improve the gait ability of patients with ASD, Yagi et al. note that postsurgical gait function still does not match the performance of age- and sex-matched healthy controls [38]. Moreover, this study highlighted the importance of the pre-operative assessment of balance, coordination, and perhaps dual tasking in patients undergoing adult spinal deformity correction. Our data showed that patients with baseline balance disturbances were less likely to adapt to their new alignment given through the construct, proven by the higher rate of PJK and implant-related complications. This corroborates with Glassman et al., who reported a 76% prevalence of undiagnosed neurologic disorders in their 29 cases of PJK [41].

With a growing emphasis on pre-operative risk stratification, it is important to identify which patients are at higher risk of operative complications. ASD surgery is complex and can be associated with substantial post-operative morbidity, including medical complications and the need for revision surgery [42,43]. PJK is a common complication after ASD surgery which can present with pain and, in severe cases, neurological deficits [44]. Prior research has examined risk factors for PJK, including age, fusion to ilium, and changes in radiological parameters [45]. Miller et al. also revealed that frailty is an independent predictor of PJK in patients undergoing ASD surgery [46]. Lafage et al. virtually modeled the risk factors for PJK following ASD surgery [47]. The authors demonstrated that the virtual modeling technique had strong correlations with actual post-operative alignment and that post-operative PJK may develop in part as a compensatory mechanism to over-correct for sagittal deformities. These findings highlight the need not only for accurate surgical planning to avoid post-operative PJK, but also the need to identify ideal patient-specific alignment parameters to avoid complications and achieve the best outcomes. Scheer et al. used the ISSG database to develop a pre-operative predictive model for major complications following ASD surgery [48]. The developed model demonstrated an overall accuracy of 87.6%, indicating a very good model fit. Twenty variables were determined to be the top predictors, including age, the number of decompression levels, number of interbody fusion levels, Scoliosis Research Society (SRS)-Schwab coronal curve type, Charlson Comorbidity Index (CCI), American Society of Anesthesiologists (ASA) grade, presence of osteoporosis, pelvic tilt, sagittal vertical axis, primary versus revision surgery, and pelvic incidence–lumbar lordosis mismatch. These findings indicate that a predictive model for major complications can be developed and has the potential to provide improved education and decision making for surgeons and patients considering ASD surgery. The present study furthers our understanding of PJK risk factors by identifying 2.2-times-increased odds of PJK in patients who report imbalance, with increased risk of other implant-related complications. Further research is needed to appreciate how this group of patients can be optimized pre-operatively with targeted interventions. However, one must note the low R^2^ of the model, demonstrating a weak relationship between the model and its dependent variable.

Our results highlight the importance of pre-operative functional assessment in ASD patients [23]. This can be performed using functional movement tests (FMTs). Standardized FMTs are usually easy to perform and only require a stopwatch, a stool, and a chair. The sit-to-stand test (STS), the alternate step test (AST), the 6-meter-walk test (SMT), and the timed up-and-go test (TUGT) are the easiest tests to administer in a clinical setting [49,50,51,52,53]. According to Lee et al., compared to lumbar stenosis patients, ASD patients required substantially more time to complete the majority of these tests [54]. This could be explained by the deformity group’s positive SVA pushing the gravity line forward and eventually producing a compensatory crouched gait. All four FMTs in the ASD group showed a substantial correlation with ODI, in contrast to the stenosis group. Only STS, however, showed a correlation with the European Quality of Life Five Dimension (EQ-5D), supporting an earlier discovery by Severijns et al. and making it a more accurate measure of quality of life [54,55]. Diebo et al. established a protocol for evaluating global functional capabilities using the Dubousset functional test (DFT) [56]. The steps exam, the down and sitting test, the up and walking test, and the dual-tasking test are the four components of this assessment. Diebo et al. discovered a correlation between DFT performance time and cognitive ability scores, such as ODI, SF12-MCS, SF12-PCS, and Montreal cognitive assessment (MoCA), when applied to individuals with ASD and degenerative lumbar disease [57]. The same team’s additional research revealed that patients who took longer to complete the Dubousset test had more severe sagittal malalignment and had lower PROMS (EQ5D, ODI, and PCS) [58].

There is an increasing interest in quantifying balance performance using clinical scales as a complement to these FMTs. Based on the balance evaluation systems test (BESTest) and the trunk control measurement scale (TCMS) tests, Severijns et al. developed and validated the functional assessment scale for spinal deformity (FASD). With exceptional intra- and inter-rater reliability, the FASD assesses balance as well as global function. In ASD patients with sagittal deformity but not coronal deformity, the FASD substantially correlated with radiographic measurements and patient-reported outcomes [59]. Furthermore, there was a higher association with PROMs than with radiographic sagittal characteristics [59]. This result emphasizes even more how important it is to evaluate dynamic parameters in order to fully assess a patient with ASD’s functional status.

Although a lot of research has been published on the spinopelvic alignment of patients with ASD based on radiographic parameters and PROMs, the dynamic aspect of this condition has not received enough attention. Recent research has addressed this gap by using 3D motion analysis to provide a more thorough assessment of ASD patients in addition to static radiographic measures and PROMs. The quantification of kinematics and kinetics during routine actions, including walking, standing, getting up from a sitting position, and using stairs, is made possible by three-dimensional motion analysis. In addition to spatiotemporal characteristics (such as walking speed, cadence, and step length), it gives a set of kinematic parameters by computing joint and segment angles in the three planes throughout the performed activity. During motion acquisition, force plates can also be employed to analyze forces and moments in the joints. Using surface electrodes, a dynamic electromyogram can assess muscle activity during movement. In conclusion, this method offers a thorough understanding of both the typical biomechanics of asymptomatic individuals and the changes that transpire in the context of disease, permitting a thorough understanding of the influence of spinal pathologies on a patient’s functional state [23].

In fact, gait analysis was used by Yagi et al. to assess ASD patients both before and after surgery [38]. ASD patients had pre-operative differences from control participants in terms of hip range of motion, shorter and asymmetric strides, and slower walking speeds. After the procedure, these deficits considerably improved, although they did not return to the control participants’ levels. In order to further address gait impairment in individuals with ASD, the authors recommended post-operative muscle strengthening. These results were confirmed by Kawkabani et al., who observed that patients with ASD walked more slowly and had shorter strides [24]. They also reported a negative correlation between these variables and the degree of knee flexion, SVA, and PT on static radiographs [38]. Later, Mekhael et al. showed that in a machine learning simulation, dynamic measures outperform radiographic measures in terms of their ability to predict HRQOLs [60]. Surprisingly, their research revealed that the sole use of dynamic measures was superior to combining radiographic and dynamic measures in terms of prediction accuracy [60]. This emphasizes how crucial it is to use gait analysis in order to completely understand how ASD affects a patient’s functional status.

There are several limitations of this study to consider. Firstly, imbalance and gait instability are self-reported by the patient, and therefore there is a lack of objective assessment by clinician examination or gait analysis. Therefore, one can use one of the proposed FMTs. Secondly, it is unknown if the imbalance patients have neurological conditions responsible for their loss of balance or gait instability which are not caused by ASD. This could be delineated by a more focused screening Thirdly, the PJK in imbalanced patients may or may not have been caused by trauma due to the falls; their frailty, which was shown to be higher pre-operatively; or other unknown confounding factors that might increase both the risk of falls and PJK. This could justify the weak R^2^ value of the model.

## 5. Conclusions

Patients with a self-reported loss of balance and unsteady gait have significantly worse baseline frailty and PROMs, as compared to those without reported imbalance. Although surgical intervention improves the sagittal and coronal alignment, imbalance patients have increased rates of PJK and implant-related complications. Asking patients about balance and falls may serve as a simple yet effective proxy for pre-operative risk stratification. However, implementing FMTs and more sophisticated functional testing may be better and more targeted for ASD patients.

## Figures and Tables

**Table 1:** Demographics of imbalance and balance cohorts.

Mean (+/−SD)	Imbalance (*n* = 106)	Balance (*n* = 106)	*p*-Value
Age	64.21 (±9.80)	63.03 (±9.07)	0.36
Gender (% Female)	76%	87%	0.052
BMI	27.22 (±4.89)	26.99 (±4.60)	0.73
Frailty Index	3.74 (±1.33)	2.33 (±1.22)	<0.001
Median UIV	T9	T8	0.55
Median LIV	Ilium	Ilium	0.73

**Table 2:** Radiographical profile of imbalance and balance groups at baseline.

Mean (+/−SD)	Imbalance	Balance	*p*-Value (Cohen’s d)
Sagittal Profile			
pelvic tilt	24.16° (±9.23)	24.66° (±9.20)	0.69 (0.05)
Sagittal Vertical Alignment	53.18 mm (±58.11)	51.71 mm (±53.23)	0.85 (0.03)
PI-LL	13.60° (±16.38)	15.10° (±16.75)	0.51 (0.09)
T4-T12 kyphosis	34.76° (±15.78)	32.17° (±17.60)	0.26 (0.15)
T1-PA	21.78° (±11.25)	21.74° (±9.94)	0.97 (0.003)
Coronal Profile			
Upper Thoracic Cobb Angle	16.02° (±9.21)	18.51° (±10.09)	0.17 (0.26)
thoracic cobb angle	25.27° (±16.54)	37.45° (±22.01)	<0.001 (0.64)
Thoracolumbar Cobb Angle	39.77° (±23.30)	39.64° (±26.26)	0.98 (0.005)
Lumbar Cobb Angle	37.03° (±16.74)	45.53° (±18.37)	0.004 (0.46)
C7 PLA (Global Coronal Balance)	41.51 mm (±43.68)	34.25 mm (±28.93)	0.15 (0.20)

**Table 3:** Patient-reported outcome measures at baseline.

Mean (+/−SD)	Imbalance	Balance	*p*-Value (Cohen’s d)
ODI	45.15 (±16.56)	36.62 (±14.19)	<0.001 (0.6)
SF-36—physical component score	30.17 (±8.73)	35.10 (±9.95)	<0.001 (0.5)
SF-36—mental component score	44.04 (±12.65)	51.76 (±10.32)	<0.001 (0.6)
SRS—activity	2.75 (±0.78)	3.29 (±0.80)	<0.001 (0.8)
SRS—pain	2.26 (±0.80)	2.73 (±0.78)	<0.001 (0.6)
SRS—appearance	2.41 (±0.68)	2.71 (±0.70)	0.02 (0.4)
SRS—mental	3.28 (±0.85)	3.80 (±0.80)	<0.001 (0.6)
SRS—satisfaction	2.71 (±1.04)	2.83 (±1.00)	0.40 (0.1)
total SRS score	2.68 (±0.56)	3.11 (±0.54)	<0.001 (0.9)

**Table 4:** Correction from baseline to 2 years post-operative.

Mean (+/−SD)	Imbalance	Balance	*p*-Value (Cohen’s d)
Sagittal Profile			
pelvic tilt	−1.45° (±7.91)	−3.60° (±7.16)	0.04 (0.3)
Sagittal Vertical Alignment	−29.23 mm (±56.32)	−35.67 mm (±53.52)	0.39 (0.1)
PI-LL	−11.93° (±15.79)	−15.08° (±15.47)	0.14 (0.2)
T1PA	−3.22° (9.33)	−5.77° (9.28)	0.047 (0.3)
Coronal Profile			
Maximum Cobb Angle Correction	−26.51° (14.89)	−29.36° (14.90)	0.167 (0.2)
Upper Thoracic Cobb Angle	−3.57° (±7.54)	−4.31° (±7.66)	0.62 (0.1)
thoracic cobb angle	−11.41° (±10.65)	−16.68° (±13.75)	0.01 (0.4)
Thoracolumbar Cobb Angle	−21.99° (±16.89)	−20.52° (±16.51)	0.62 (0.1)
Lumbar Cobb Angle	−22.53° (±12.66)	−26.41° (±14.86)	0.09 (0.3)
C7 PLA (Global Coronal Balance)	−11.24 mm (±42.18)	−7.66 mm (±30.51)	0.48 (0.1)

**Table 5:** Complications of imbalance vs. balance groups.

Mean (+/−SD)	Imbalance	Balance	*p*-Value
re-operation	29.2%	30.2%	0.88
major complication	23.6%	30.2%	0.28
fusion achieved after 2 years	60.4%	64.2%	0.57
PJK *	26.4%	14.2%	0.03
PJF	34.00%	17.92%	0.01
implant-related complications	47.2%	34.0%	0.05

* Logistic regression (controlling for age, baseline sagittal parameters, PI-LL correction, and CCI): imbalance patients had OR = 2.2 of sustaining PJK (*p* = 0.03, r^2^ = 0.024).

## Data Availability

The data associated with our study have not been deposited into a publicly available repository. They are available upon reasonable request.
